# Traumatic Brain Injury Recovery with Photobiomodulation: Cellular Mechanisms, Clinical Evidence, and Future Potential

**DOI:** 10.3390/cells13050385

**Published:** 2024-02-23

**Authors:** Lew Lim

**Affiliations:** Vielight Inc., Toronto, ON M4Y 2G8, Canada; lewlim@vielight.com

**Keywords:** traumatic brain injury, photobiomodulation, pathophysiology, cellular mechanisms, clinical studies, parameters, power, pulse frequency

## Abstract

Traumatic Brain Injury (TBI) remains a significant global health challenge, lacking effective pharmacological treatments. This shortcoming is attributed to TBI’s heterogeneous and complex pathophysiology, which includes axonal damage, mitochondrial dysfunction, oxidative stress, and persistent neuroinflammation. The objective of this study is to analyze transcranial photobiomodulation (PBM), which employs specific red to near-infrared light wavelengths to modulate brain functions, as a promising therapy to address TBI’s complex pathophysiology in a single intervention. This study reviews the feasibility of this therapy, firstly by synthesizing PBM’s cellular mechanisms with each identified TBI’s pathophysiological aspect. The outcomes in human clinical studies are then reviewed. The findings support PBM’s potential for treating TBI, notwithstanding variations in parameters such as wavelength, power density, dose, light source positioning, and pulse frequencies. Emerging data indicate that each of these parameters plays a role in the outcomes. Additionally, new research into PBM’s effects on the electrical properties and polymerization dynamics of neuronal microstructures, like microtubules and tubulins, provides insights for future parameter optimization. In summary, transcranial PBM represents a multifaceted therapeutic intervention for TBI with vast potential which may be fulfilled by optimizing the parameters. Future research should investigate optimizing these parameters, which is possible by incorporating artificial intelligence.

## 1. Introduction

Traumatic Brain Injury (TBI) poses a significant public health challenge and is predominantly instigated by an external mechanical force. It is a leading cause of mortality and long-term disability globally, with annual incidences estimated between 64 and 74 million cases [1]. Clinically, TBI manifests through a spectrum of symptoms ranging from coma and headache to behavioral dysfunctions such as aphasia, seizures, amnesia, aggression, and anxiety [2,3].

Currently, there are no FDA-approved pharmacotherapies for TBI recovery. TBI pathogenesis involves a myriad of post-injury responses and secondary damage to brain tissues, culminating in cellular damage and loss [4].

This absence of effective disease-modifying treatments is partly attributable to TBI’s intricate pathophysiology, suggesting the necessity for multifaceted therapeutic strategies. The resultant structural and functional deficits can be permanent, underlined by complex pathophysiological and cellular mechanisms [5].

Given these complexities, conventional single-modality interventions are unlikely to be effective. Success in TBI treatment is anticipated to stem from innovative approaches, underpinned by multimodal diagnostic techniques [6]. The mechanisms of photobiomodulation (PBM) directly and indirectly identify with many cellular mechanisms associated with the complex TBI pathophysiology [7].

In this context, transcranial PBM emerges as a promising intervention. PBM, involving the application of red and/or near-infrared (NIR) light to the brain transcranially or intranasally, has shown the potential to expedite recovery from TBI symptoms and mitigate associated symptoms. Clinical study evidence, both published and unpublished, indicates PBM’s efficacy across various TBI severities, including chronic traumatic encephalopathy (CTE) [8].

Notably, the mechanisms and physiological processes underpinning PBM have been extensively explored. Evidence suggests that PBM exhibits antiapoptotic, anti-inflammatory, and pro-proliferative effects, in addition to modulating cellular metabolism [9]. New discoveries and ongoing research on cellular research will contribute to improved outcomes.

**Figure 1:**
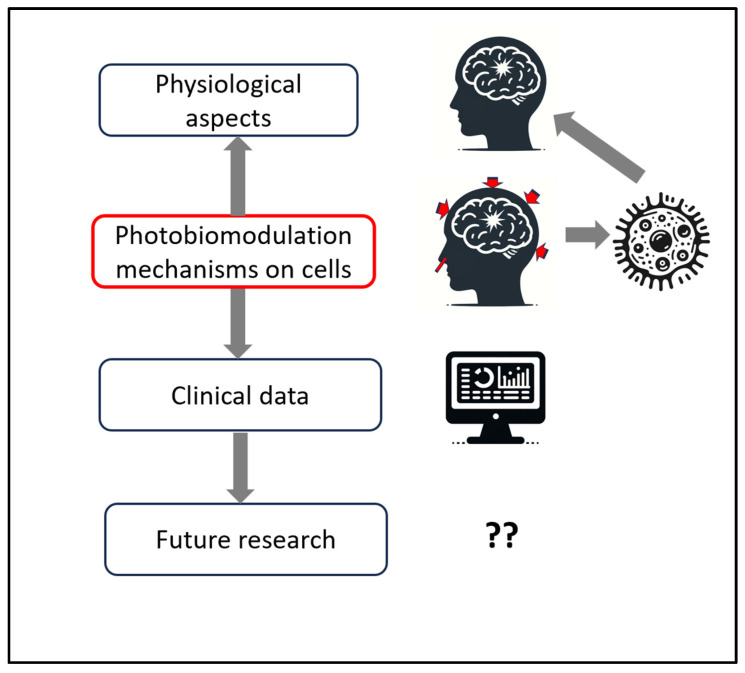
Schematic structure of the manuscript’s reviews and discussion, starting with a review of the pathophysiological aspects of traumatic brain injury (TBI), matching with photobiomodulation (PBM) research on cellular mechanisms, supported by clinical data in the literature, and ending with discussions on future research for parameters to improve outcomes for TBI.

Figure 1 provides a schematic structure of the reviews and discussion in this manuscript.

## 2. Pathophysiological Aspects of TBI and Related PBM Research

The pathophysiological aspects of TBI can be grouped into axonal injury, excitotoxicity, mitochondrial dysfunction, release of reactive oxygen species and oxidative stress, neuroinflammation, axonal degeneration and growth inhibitors, apoptotic cell death, and dysfunctional autophagy [4].

**Figure 2:**
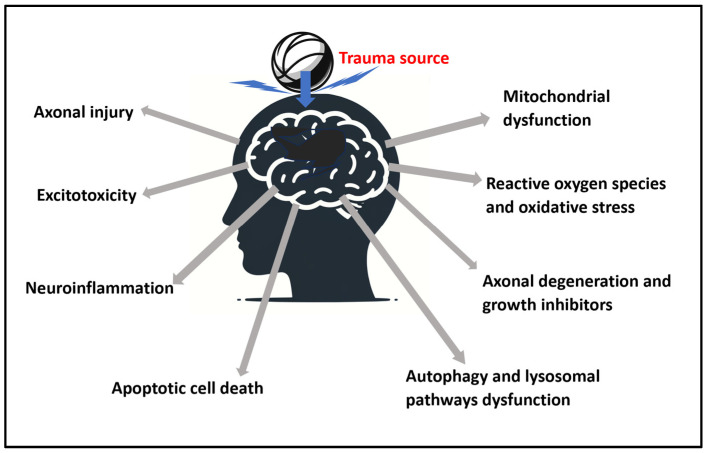
Summary of the identified pathophysiological aspects of traumatic brain injury (TBI) from a trauma source that are addressable with photobiomodulation (PBM).

A summary presentation of the physiological aspects is presented in Figure 2.

For each of these, we can also identify cellular molecular mechanisms activated by PBM to address them.

For this section, we searched online and the Google Scholar and PubMed databases for PBM studies that were available until 31 December 2023 and investigated cellular mechanisms related to the abovementioned pathophysiological aspects. We then reviewed the findings to evaluate PBM effects and unveil the opportunities for translation into beneficial applications. The results for this section are summarized in Table 1, and details are presented below.

### 2.1. Axonal Injury

Axonal injury, a primary pathological feature of TBI, correlates with injury severity. Typically, TBI results in diffuse axonal injury (approximately 70%) due to coup and contre-coup forces, leading to layered brain hemorrhages [10].

PBM may aid in repairing axonal damage by increasing the production of cellular energy currency, adenosine triphosphate (ATP). This process involves the regulation of various secondary mediators: reactive oxygen species (ROS), nitric oxide (N), cyclic adenosine monophosphate (cAMP, and calcium ions (Ca^2+^). These mediators play key roles in cellular signaling and function, and their modulation by PBM can lead to the activation of pathways that promote the regeneration of axons [11].

A study conducted on rats showed that treatment with PBM significantly helped repair nerve fibers. This effect was associated with an increase in the activity of certain enzymes in utilizing the ATP—phosphoinositide 3-kinases/protein kinase B (PI3K/Akt) cellular signaling pathway, crucial for many cell functions, including energy management and cell growth [12].

The other pathophysiological aspects below are considered secondary to axonal injury but are vital to the health of the post-TBI brain.

### 2.2. Mitochondrial Dysfunction

The functions of mitochondria include oxidative phosphorylation, where oxygen is used to produce ATP. They also have important roles in ion homeostasis, several metabolic pathways, apoptosis and programed cell death, and ROS production and consumption [13]. The role of these physiological aspects, and the need to balance them are expanded below. The dysfunction of the mitochondria in managing these are linked to many neurological conditions [14]. At the cellular level, the main cause of secondary harmful cascades is cell damage centered in the mitochondria [15].

Electron microscopy studies of mitochondria post-TBI have shown significant structural damage, such as swelling, disruption of cristae (internal structures of mitochondria), and loss of membrane potential, all of which indicate impaired mitochondrial function. In the process, proteins such as cytochrome c and apoptosis-inducing factor (AIF) are released into the cytosol, leading to further cell death [16,17].

PBM’s effectiveness is often attributed to its action on mitochondria, particularly cytochrome c oxidase (CCO) in the electron transport chain (ETC), playing a key role in enhancing oxidative phosphorylation [18]. It is hypothesized that inhibitory nitric oxide can be dissociated from CCO. This is part of the process of PBM enhancing ETC activity, mitochondrial membrane potential, and promoting cellular recovery [19].

Through its action on the mitochondria, PBM can aid in cellular recovery by improving energy production, reducing oxidative stress, potentially mitigating apoptosis, and promoting axonal regeneration [11].

### 2.3. Excitotoxicity

The blood–brain barrier (BBB) is a protective barrier that regulates the movement of substances between the bloodstream and the brain. TBI-induced BBB breakdown leads to excessive excitatory amino acid release, particularly glutamate, resulting in oxidative stress and prolonged excitotoxicity [20].

Excitotoxicity occurs when glutamate over-activates its receptors, such as N-methyl-D-aspartate (NMDA) and kainate (KA) receptors, leading to an influx of calcium ions (Ca^2+^) into neurons. Excessive glutamate during TBI is also attributed to a failure of glutamate re-uptake due to the dysfunction of glutamate transporters [21]. The excess Ca^2+^ can trigger a cascade of harmful events, including oxidative stress and neuronal damage [20].

While studies show PBM increases Ca^2+^ concentration in healthy cells, PBM also plays a homeostatic role in moderating the toxic levels of intracellular Ca^2+^ content in stressed cells. In an in vitro study, excitotoxicity was induced in cultured cortical neurons with glutamate, N-methyl-D-aspartate (NMDA), and kainic acid (KA) to produce 50% cytotoxicity. The effects of PBM on these excitotoxic cells were compared against a control of healthy neurons. Compared with the control, samples treated with PBM at 810 nm had increased intracellular Ca^2+^ in healthy neurons but decreased significantly in the excitotoxic neurons [22].

In summary, TBI pathological effects on the BBB and subsequent excitotoxicity due to glutamate overload, lead to neuronal damage through oxidative stress. PBM appears to counter these effects by enhancing cellular energy production, regulating Ca^2+^ concentrations, and improving mitochondrial health, thereby offering a potential therapeutic strategy to mitigate the damaging effects of TBI-induced excitotoxicity.

### 2.4. Reactive Oxygen Species, Reactive Nitrogen Species, and Oxidative Stress

Reactive oxygen species (ROS) and reactive nitrogen species (RNS) are generated as natural byproducts of the normal metabolism of oxygen. At low levels, ROS is essential for the regulation of physiological cellular functions, including cellular signaling, differentiation, and survival [23,24]. RNS has been recognized as playing a crucial role in the physiologic regulation of many, if not all, living cells, including nervous system cells [25].

TBI causes an overproduction of ROS and RNS which can overwhelm the brain’s antioxidant defenses. This leads to oxidative stress, a state where the body’s ability to detoxify these excess reactive products is disrupted. Oxidative stress results in damage to cell components, including lipids, proteins, and DNA. This can cause mutations, malfunctions in cellular signaling pathways, and can initiate programmed cell death (apoptosis), contributing to chronic traumatic encephalopathy (CTE) neurodegeneration and other diseases [26,27].

PBM generally produces ROS at low levels, resulting in positive effects [28]. PBM has been shown to modulate ROS levels positively, increasing antioxidant enzyme activity in superoxide dismutase (SOD), catalase (CAT), and glutathione peroxidase (GPx), leading to the reduction of oxidative stress in brain tissues [29,30]. Injured cells subjected to high levels of oxidative stress, such as in the case of TBI, are also more responsive to PBM. It has been found that the more stressed the cells are, the better they respond to PBM [31]. The response tends to decrease the intracellular concentration of ROS [32].

Near infrared (NIR) PBM has been found to regulate the production of ATP and ROS in mitochondria. Interestingly, in isolated mitochondria, a 980 nm diode laser wavelength at 100 mW decreases ATP and increases ROS production, whereas 800 mW and above increases ATP and reduces ROS production [33]. The relatively higher power was shown to be more inhibitive to ROS.

Another group experimented with a different set of parameters using a 635 nm laser on skin fibroblasts cultured in high glucose to create high ROS levels. They found that PBM can increase the antioxidant capacity of cells by reducing the high concentration of ROS induced by high glucose [30].

A systematic review of PBM used in healthy human subjects analyzed the effects of PBM on exercise-induced oxidative stress compared with placebo therapy. It was concluded that PBM can reduce oxidative damage and increase enzymatic antioxidant activity after exercise, and one session can modulate the redox mechanism [34].

In an experiment, Macedo et al. surmised that PBM therapy increased the activity of glutathione enzymes (GST and GPX), which are highly important for the protection of the lung against oxygen and nitrogen reactive species (RONS) [35].

In summary, TBI involves the overproduction of RONS leading to cellular damage. PBM can reduce the incidence of high levels of RONS and consequential oxidative stress. A large part is contributed by PBM’s enhancement of antioxidative activity.

### 2.5. Neuroinflammation

In response to TBI, glial cells, including microglia and astrocytes, become activated and release inflammatory mediators, contributing to the inflammatory response [36]. TBI can lead to a breakdown or disruption of the BBB, further facilitating the infiltration of immune cells and substances that can exacerbate inflammation and neuronal damage [37] Excessive or prolonged glial activation can cause neurotoxicity, synaptic dysfunction, and neurodegeneration [38].

A study published in 2023 used a mouse model to investigate inflammation induced by lipopolysaccharide (LPS), a component found in the outer membrane of certain bacteria known to trigger an immune response. The study demonstrated PBM’s efficacy in downregulating proinflammatory cytokines (IL-1β and IL-18). These cytokines are signaling molecules that promote inflammation and are typically elevated after TBI. In the meantime, PBM was also shown to upregulate anti-inflammatory cytokines (IL-10). These molecules help in reducing inflammation and promoting healing. The study observed improvements in cognitive performance in the mice, suggesting that PBM might mitigate some of the cognitive deficits associated with neuroinflammation [39].

In other animal models studying PBM effects on TBI, improvements were observed for ischemia, neurodegeneration, aging, epilepsy, depression, and spinal cord injury. PBM is therefore a promising modality to treat brain pathological conditions induced by neuroinflammation [9].

In summary, PBM has been shown to reduce inflammation in the brain, which is a common response to TBI. It can modulate the activity of pro-inflammatory cytokines and promote the release of anti-inflammatory cytokines. By reducing inflammation, PBM may help mitigate secondary damage caused by excessive immune responses [40].

### 2.6. Axonal Degeneration and Growth Inhibitors

In TBI, axons, which are responsible for transmitting information in the nervous system, can become damaged or severed. This damage can lead to axonal degeneration, disrupts neural communication, and can be responsible for brain swelling and neuronal death [41]. Axonal degeneration can trigger post-traumatic neurodegeneration, presented with toxic protein pathologies amyloid and hyperphosphorylated tau. These protein pathologies are found in neurodegenerative diseases such as Alzheimer’s and Parkinson’s. In the case of TBI, its equivalent is the presence of CTE [42,43].

After TBI, the brain can produce molecules known as growth inhibitors [4]. These molecules impede the ability of neurons to regenerate axons, thus hindering the repair process. Glial cells, such as astrocytes and microglia, may contribute to this process by forming a scar tissue barrier around the injury site, which releases inhibitory molecules preventing axonal regrowth. They can also activate downstream effectors that lead to axonal collapse [44].

PBM has been shown to promote axonal regeneration. This could be due to PBM’s effects on mitochondrial function in neurons, enhancing their energy production and survival, which are crucial for repair and regeneration processes [11]. In a controlled animal study of lesioned facial nerve treated with PBM, the nervous function was restored in 30 days with PBM treatment. This was contrasted with the control group which showed no recovery, indicative of the effect of PBM for axonal regeneration following nerve damage [45]. Another study investigated the effects of PBM on neuronal axon regeneration under oxidative stress in vitro. This shows that PBM reduced ROS levels, increased neuronal survival, and promoted axonal growth and branching [46].

In summary, the mechanisms of action behind PBM’s effectiveness in countering TBI-related axonal degeneration involve enhancing the regenerative capacity of axons and reducing the inhibitory environment created by growth inhibitors and inflammation. This is supported by evidence from research on spinal cord injury and oxidative stress models, which show PBM’s potential in promoting neural repair and reducing secondary damage.

### 2.7. Apoptotic Cell Death

TBI can trigger a form of programmed cell death, apoptosis, in both neurons and glial cells. This process involves a series of biochemical events leading to characteristic cell changes and death. It involves mitochondrial dysfunction, caspase activation, DNA fragmentation, and phagocytosis [47]. The loss of neurons and glial cells through apoptosis significantly contributes to the loss of brain function. It can also induce secondary inflammatory cascades that can exacerbate brain damage [48].

PBM has shown potential in reversing the apoptotic process. This effect is believed to be mediated through its action on cellular mitochondria [11]. PBM can activate cell survival pathways involving various signaling molecules and proteins that work together to prevent the cell from undergoing apoptosis [19].

In addition to its role in inhibiting cell death, PBM has also been observed to stimulate neurogenesis, the process of generating new neurons from neural stem cells. An animal study demonstrated that PBM can enhance neurogenesis following injuries similar to ischemic stroke. The proposed mechanisms underlying this effect include (1) the promotion of proliferation and differentiation of neural progenitor cells within the peri-infarct zone, which is the area surrounding the damaged brain tissue; and (2) the improvement of the neuronal microenvironment. This improvement is achieved by modulating the inflammatory status and enhancing mitochondrial function [49].

Angiogenesis plays a crucial role in supporting neurogenesis during the recovery from TBI. Specifically, angiogenesis, stimulated by vascular endothelial growth factor (VEGF), has been shown to significantly enhance neurogenesis and reduce lesion volumes post-TBI [50]. This process is also critical in remodeling neurogenesis following ischemic strokes [51]. PBM has demonstrated efficacy in promoting angiogenesis. It does this by modulating endothelial dysfunction [52] and aiding in wound healing [53], serving as examples of its angiogenic effects.

In the context of brain injury in human cases, the generation of new neurons can aid in recovery and functional restoration. In this respect, Chao et al. reported a TBI case involving a 23-year-old professional hockey player with a history of concussions, with symptoms of headaches, mild anxiety, and difficulty concentrating. He treated himself with a PBM device for 8 weeks. MRI showed increased brain volumes together with improved functional connectivity, increased cerebral perfusion, and improvements in neuropsychological test scores [54]. This suggests that PBM may help in reducing cell death and enhancing brain repair mechanisms.

In an animal study involving a mouse model of lipopolysaccharide-induced inflammation, a significantly higher proportion of apoptotic cells was observed in the control group than in the PBM group. This means that PBM has the potential to reduce cell loss to apoptosis in injured neurological tissue [55].

Cell culture studies provide explanations of PBM-induced anti-apoptosis based on identified protein modifications decreasing an apoptosis-regulating protein and suppressing the activity of components for efficient apoptosis [56].

In summary, PBM can support the survival of neurons and other brain cells following TBI. It can activate prosurvival pathways and anti-apoptotic mechanisms, potentially preventing cell death in injured brain tissue [57]. Studies suggest that PBM stimulates neurogenesis, the process of generating new neurons from neural stem cells. This is particularly relevant for chronic TBI cases in which cognitive and neurological deficits persist due to cell death [47,54]. PBM as a multifaceted approach not only helps in preventing further cell loss but also contributes to the restoration of brain function, as demonstrated in both human case studies and animal models.

### 2.8. Autophagy and Lysosomal Pathways Dysfunction

Autophagy is a cellular process that involves the degradation and recycling of damaged or unnecessary cellular components. It plays a crucial role in maintaining cellular homeostasis and health. Lysosomes are cellular organelles that contain enzymes to break down waste materials and cellular debris and are integral to the autophagy process. Autophagy and lysosomal function are essential for maintaining cellular homeostasis, clearing damaged or unwanted materials, and regulating cellular metabolism [58].

TBI can disrupt these processes, impairing the ability of cells to efficiently remove and recycle damaged components. This disruption can lead to the accumulation of toxic proteins and cellular components, contributing to cell death and exacerbating brain damage [59,60].

PBM is hypothesized to regulate ROS levels, thereby facilitating healthy mitophagy and maintaining cellular homeostasis. Mitophagy is a specific type of autophagy focused on the degradation and recycling of mitochondria. It helps to support the brain which is impaired during TBI [61] as a result of mitochondrial dysfunction [15]. By regulating ROS levels, PBM may enhance mitophagy, helping cells to remove dysfunctional mitochondria [62]. This in turn supports protein activity (involving PINK1/Parkin signaling) [63], which helps regulate mitophagy [64].

In summary, TBI impairs autophagy and lysosomal functions, leading to toxic protein build-up and cell death. PBM is hypothesized to help counteract these effects by modulating ROS levels through mitochondrial activity, thus facilitating healthy mitophagy and helping to restore cellular homeostasis [56]. This is particularly important in the context of brain injury, where cellular health is critical to recovery and function.

**Table 1:** Summary of pathophysiological aspects, cellular mechanisms, and related photobiomodulation research.

Pathophysiological Aspects	Description of the PBM Research
Axonal Injury	PBM may aid axonal recovery through improved ATP generation and modulation of secondary mediators. It activates the PI3K/Akt signaling pathway [11,12].
Mitochondrial Dysfunction	The effects of PBM on mitochondria, particularly cytochrome c oxidase, can restore electron transport and increase mitochondrial membrane potential, aiding axonal regeneration [11,19].
Excitotoxicity	PBM was found to increase ATP content, Ca^2+^ levels, and mitochondrial membrane potential, counteracting excitotoxicity [22].
Reactive oxygen and nitrogen species (RONS)	PBM reduces RONS levels and oxidative stress, promoting antioxidant capacity and reducing damage. It can modulate exercise-induced oxidative stress [30,32,33,34,35].
Neuroinflammation	PBM can reduce pro-inflammatory cytokines, activate anti-inflammatory responses, and downregulate neurotoxic microglia and astrocytes [9,39,40].
Axonal Degeneration	PBM increases axonal regeneration and counters growth inhibitors, potentially preventing axonal damage and degeneration [4,44,45,46].
Apoptotic cell death	PBM activates anti-apoptotic mechanisms, potentially preventing cell death and promoting neurogenesis [47,54,55,56,57].
Autophagy and Lysosomal Dysfunction	PBM can restore mitochondrial function and improve mitophagy by regulating autophagy and lysosomal activity [61,62,63,64].
Additional Systemic Mechanisms	PBM enhances cellular energy production, improves blood flow, modulates synaptic plasticity, and reduces ferroptosis [65,66,67,68,69,70].

In addition to the above, the following systemic and secondary PBM mechanisms are also applicable to treating TBI.

## 3. Additional Relevant Systemic and Secondary PBM Mechanisms 

Certain PBM mechanisms have systemic effects, with availability across the different pathophysiological elements related to TBI.

### 3.1. Increased Cellular Energy Production

In PBM, when photons from the light source interact with cytochrome c oxidase in mitochondria, it can lead to increased ATP production. This enhanced energy production improves cellular function and repairs damaged brain tissues [71].

### 3.2. Enhanced Blood Flow and Oxygenation

PBM is believed to enhance cellular energy availability by improving blood circulation through the photodissociation of nitric oxide (NO). This improves blood flow and oxygen delivery to the injured brain region. It promotes tissue repair and reduces hypoxic conditions that can exacerbate TBI-related damage [65]. A 2016 published animal study suggested that 660 and 810 nm wavelengths pulsing at 10 Hz produced the best outcomes in TBI by improving blood flow and oxygenation [66].

### 3.3. Modulation of Synaptic Plasticity

PBM may influence synaptic plasticity, which is the ability of synapses to strengthen or weaken over time, affecting neuronal signaling. By promoting synaptic plasticity, PBM could enhance cognitive recovery and functional improvements in TBI patients [67,68].

The above literature on the effects of PBM on the pathophysiology of TBI shows the promise of PBM for treating TBI. The real value will lie in the translation to human use, as confirmed by clinical study data.

### 3.4. Effect on Ferroptosis

Ferroptosis can play a significant role in neuronal death and brain damage following the injury [72]. It is a form of regulated cell death characterized by iron-dependent lipid peroxidation linked to oxidative stress and inflammation [69]. PBM has been observed to reduce oxidative stress [70] and modulate inflammatory responses [39], which could influence ferroptosis pathways.

## 4. Clinical Data on PBM Effects on Human TBI

For many years, outcome data have been based on some animal studies. The average volume of the mouse brain is approximately 0.4 cm^3^, while the average volume of the human brain is approximately 1400 cm^3^ [73]. This means that the human brain is approximately 3500 times larger than the mouse brain in terms of volume, underlining the fact that effective parameters for the mouse model will not be the same for a human. Therefore, while animal models provide valuable early information on brain functions and safety, we should now focus on human studies for greater relevance.

In this section, a qualitative review was undertaken to gain insights aimed at enhancing the use of PBM for TBI recovery. The scope of this review encompassed solely human clinical studies while excluding non-human research findings. An examination of available human clinical studies from the existing literature was conducted, with a primary focus on information extraction. The search process involved comprehensive online searches through the Google Scholar and PubMed databases that were available as of 31 December 2023. It should be noted that the limited number of accessible human studies, coupled with significant disparities in research methodologies, utilized devices, and measurement parameters, rendered the application of traditional quantitative comparative analyses impractical. Consequently, traditional tests of bias, comparative effect sizes, and other statistical measures were not used. In contrast, the chosen qualitative review methodology seeks to provide a contextual understanding that can furnish valuable insights, thereby potentially informing the optimization of PBM applications for the attainment of superior outcomes in TBI treatment. With this review basis, the findings and subsequent perspectives are presented below.

The most recent systematic review on PBM-TBI studies (by Stevens et al., with data up to October 2021) [9] contained only animal studies, except for one human study involving 68 randomized subjects using a closed helmet with LEDs emitting NIR light. This is discussed in more detail below and in Figueiro Longo et al. (2020) [74].

The review did not include several small studies that showed the potential of PBM to treat TBI. All the available published studies are included in this manuscript for the purpose of extracting key findings that can inform the quest for improving treatment outcomes with PBM. These are summarized in chronological order below.

In an early 2011 report, Naeser et al. reported two human TBI cases that improved with PBM with 870 nm and 633 nm light emitting diodes (LEDs), 22.2 mW/cm^2^ power density, and with continuous wave [75].

In 2014, Naeser et al. reported the findings in an open protocol study of 11 subjects with the same set of PBM parameters. The participants had improved sleep and fewer post-traumatic stress disorder (PTSD) symptoms, if present. Participants and their families reported better ability to perform social, interpersonal, and occupational functions [76].

In 2015, Hesse et al. reported the use of low-level lasers with a wavelength of 785 nm, continuous wave, and 10 mW/cm^2^ power density over 10 weeks on four chronic patients in a state of unresponsive wakefulness or minimal consciousness and one subacute patient in the state of akinetic mutism (total of five patients). In summary, the treatment protocol improved the patients’ alertness and awareness, but epileptic fits were potential side effects [77].

Published in 2018, Hipskind et al. used a device with an array of 402 LEDs combining wavelengths of 629 nm and 850 nm, an average power density of 6.7 mW/cm^2^, and pulsing at rates of 73, 587, and 1175 Hz over 6 weeks. They treated 12 military veterans with chronic TBI over 13 weeks using SPECT for imaging. They concluded that pulsed transcranial PBMT using LEDs shows promise in improving cognitive function and regional cerebral blood flow [78].

In a study published in 2020, Figueiro Longo et al. studied 68 (35 treatment and 33 sham control) randomized, double-blind subjects with moderate TBI using a closed helmet with LEDs emitting NIR light at 810 nm, continuous wave with a power density of 36 mW/cm^2^. The study met the primary outcome measure of safety without adverse events within the first 7 days. Measuring with diffusion magnetic resonance imaging (MRI), there were significant changes in radial diffusivity at the late subacute stage (3-month time point) between the PBM and sham groups, indicating significant remyelination with PBM at that time point. The Rivermead post-concussion questionnaire (RPQ) did not reveal changes of significance. This study provides the first human evidence to date that PBM engages neural substrates that play a role in the pathophysiological factors of moderate TBI [74]. Its safety profile suggests that it is safe to explore similar types of brain PBM with other parameters to extract more significant outcomes for TBI treatment.

A single case reported in 2020 by Chao et al. was the first and only study to reveal that neurogenesis in chronic TBI recovery was possible with PBM. The subject was a 23-year-old professional hockey player with a history of concussions, which were presumed to have caused his symptoms of headaches, mild anxiety, and difficulty concentrating. He treated himself at home with a PBM with LEDs emitting 810 nm light pulsing at 10 or 40 Hz through an intranasal and four transcranial modules that targeted nodes of the default mode network (DMN) with a maximum power density of 100 mW/cm^2^. After 8 weeks of PBM treatment, increased brain volumes, improved functional connectivity, increased cerebral perfusion, and improvements in neuropsychological test scores were observed [54].

In a 2022-published report, Rindner et al. used a low level at 1064 nm, continuous wave, with irradiance of 250 mW/cm^2^ for 10 min each session over 8 weeks on 11 patients diagnosed with TBI. The study faced challenges for patient compliance because it was conducted during the COVID-19 pandemic. The findings generally lacked significant positive clinical outcomes but suggest that PBM with the methodology was well tolerated and may have the ability to produce positive cognitive and emotional benefits for individuals with TBI [79]. In addition to the use of lasers, this study delivered far more power density than preceding studies which generally used LEDs.

Chan et al. [80] published a preprint paper in 2022 that conducted a deeper data analysis on patients from the earlier published Figueiro Longo et al. study [74]. They selected 17 treated patients, 21 sham, and 23 as healthy controls and investigated the effect of PBM on resting-state connectivity in different brain regions, differentiating between acute and subacute phases. Based on their results, the authors proposed that PBM can modulate the neuronal connectivity during the transition from acute to subacute phases in TBI (2–3-week time-point) in the frontal-parietal regions. The heterogeneity of TBI posed challenges in extracting more useful conclusions on clinical outcomes [80].

In a recent 2023 publication, Naeser et al. detailed the recovery of four retired professional (American) football players from suspected CTE. This study included a home-use device using LEDs with 810 nm wavelength, pulsing at 40 Hz and delivering power density of up to 100 mW/cm^2^. The study did not compare against sham devices. However, the clinical assessments were thorough. When assessed in the laboratory after 1 month of PBM treatment, there was significant improvement in post-traumatic stress disorder (PTSD), depression, pain, and sleep. One patient discontinued narcotic pain medications and had reduced tinnitus. The first set of treatments stopped after 1 month. At 2 months post-PBM, two cases regressed. Then, home PBM resumed with a home-use device after a 2-month break. It was applied to only cortical nodes of the default mode network (DMN) over 12 weeks. Again, significant improvements resumed. There were significant increases/improvements in salience network (SN) functional connectivity (FC) over time, along with executive function, attention, PTSD, pain, and sleep. Improvements were also observed in the central executive network (CEN) FC, verbal learning/memory, and depression. Increased n-acetyl-aspartate (NAA) (related to oxygen consumption and mitochondria) was present in the anterior cingulate cortex, parallel to less pain and PTSD [8].

Liebel et al., in a study presented in 2022 and 2023, evaluated 49 former male and female athletes with a history of concussive and/or repetitive sub-concussive events. In the non-randomized study, the participants received active PBM with 810 wavelengths, pulsing at 40 Hz with up to 100 mW/cm^2^ power density, over 8 weeks. The participants demonstrated statistically significant reductions in self-reported depression, posttraumatic stress, and adjustment symptoms compared with pre-treatment levels. Sleep quality, simple reaction time, and dominant and nondominant hand grip strength improved following PBM treatment [81] This study was presented as a poster, and a peer-reviewed submission is pending approval for publication at the time of writing.

A summary of the above findings is presented in Table 2.

Key Findings:

- The common denominator is that PBM applied to the brain is safe, with no report of significant adverse effects.
- PBM shows promise for treating chronic TBI in a degenerative state, particularly for suspected CTE.
- The efficacy outcomes were inconsistent.
- Many studies were case series that lacked sham control.
- Imaging studies through diffusion and structural MRI reveal clearer objective measured outcomes than clinical studies by partially overcoming the challenging heterogeneity of TBI.
- Data based on time-course were more conclusive than across-group comparison (such as sham and severity) due to TBI heterogeneity.
- The parameters used varied widely between studies.
- The more recent studies appear to favor higher power densities; devices that pulse produce improved clinical outcomes. This indicates that parameters used in some studies were suboptimal and compromised outcomes.
- Larger randomized controlled clinical trials are required to validate the findings.
- At the ongoing pace, and with the challenges of conducting controlled human studies, it will be many years before PBM can reach consensus on optimal parameters.

For details of the parameters, please refer to the original text, which also provides detailed nuances of clinical outcomes.

In summary, the findings indicate that PBM holds promise for the treatment of TBI. This potential can be progressively realized through continuing investments in research, facilitating new discoveries in the field.

## 5. New Discoveries in Cellular Mechanisms Inform Future PBM Treatments

In the recent systematic review of the literature published in 2021 by Stevens et al specifically related to TBI, the authors concluded the PBM produces positive physiological outcomes. However, they also suggested that there is no difference between the outcomes of continuous wave and pulsed PBM, or energy delivery [9]. With more published data, a later systematic review based on data up to July 2022 on PBM of broader brain activity, the authors suggested that parameters including power density could influence mental outcomes, and that more studies need to be conducted in this respect [82]. Since that time, more investigations have been carried out by a network of collaborators, which leads to the perspective in this manuscript that while PBM has a significant effect on TBI, modifications of the underlying cellular mechanisms by adjusting certain parameters can lead to better outcomes.

We are in the quest for effective PBM treatments, not just for TBI but also for brain conditions across the board, by conducting more detailed research into the effects of different parameters on brain functions. This was spurred by evidence of electroencephalography (EEG) waveforms modified by delivery of a chosen pulse frequency of gamma at 40 Hz, published in 2019 [83]. Brains with TBI can be characterized by certain waveforms [84]. Energy delivery via power density (mW/cm^2^) has also been found to have a significant influence on brain activity and structures.

While the precise cellular and physiological mechanisms of PBM in TBI are still under investigation, several key mechanisms have emerged based on research findings:

### 5.1. Increase in Cellular Current Flow and Resistance

One of the characteristic features of a living cell is that it controls the exchange of electrically charged ions across the cell membrane [85]. PBM has been shown to allow more electrical current to flow through cells. Interestingly, PBM also in the meantime, increases cellular resistance or resilience, which is important for the functional integrity of the myelin sheaths of the axons. This was achieved with 810 nm wavelength delivered at 10 Hz [86]. Further investigation to explore the characteristics of other pulse frequencies is warranted.

### 5.2. Polymerization of Tubulins

Dimers of α- and β-tubulin polymerize to form microtubules, which are composed of 13 protofilaments assembled around a hollow core. Tubulin dimers can depolymerize as well as polymerize, and microtubules can undergo rapid cycles of assembly and disassembly [87]. PBM pulsed at 10 Hz at 810 nm demonstrated depolymerization of tubulins [86]. There is also evidence that intervention with this set of parameters can destabilize the secondary structure of the microtubules, with α-helices transitioning into β sheets [88]. As microtubules are a core component of neuronal integrity, more knowledge in this area has implications for TBI recovery and avoidance of CTE neurodegenerative progression.

### 5.3. The Significance of Pulse Frequency

More recent studies extend beyond the effects of PBM on molecular mechanisms—they offer clues that each parameter such as wavelength, power density, and pulsing rates could influence physiological outcomes. This hypothesis is supported by other investigations that have shown that pulse frequencies influence the brain response.

In 2019, Zomorrodi et al. published for the first time that inducing a certain PBM frequency in the brain can change the waveforms of the brain. The study delivered 810 nm wavelength at 40 Hz (gamma) to the hubs of the default mode network (DMN) of healthy brains and found that this increases the power of the faster oscillations of alpha, beta, and gamma, while reducing the power of the slower oscillations of delta and theta [83].

In 2023, Tang et al. [89] reported in a randomized sham-controlled study involving 56 healthy subjects that pulsed waves at 40 Hz and 100 Hz produced significantly better cognitive effects than continuous wave and sham. Like the Zomorrodi et al. study [83], they observed significant increase in the gamma waveforms, particularly with the 40 Hz delivery. Wavelengths of 660 nm and 830 nm wavelengths were used [89].

From these findings, we can summarize what is known:

1. PBM delivered to the brain influences brain function, which is explained by a variety of cellular mechanisms.
2. Pulse frequency affects brain waveforms, with EEG can inform brain states for diagnosis.

## 6. Perspective on Effective Parameters and Further Research

The reviewed evidence indicates that certain generalized parameters involving near-infrared (NIR) wavelengths and pulsing have the potential to offer benefits to individuals experiencing post-TBI symptoms. However, it underscores the necessity for further research to yield more predictable and efficacious clinical outcomes. Generalizing with a simplified protocol is anticipated to be particularly challenging, given the inherent variability among individual subjects. A potential solution to this challenge lies in finding the ideal personalized parameter settings.

Research based on healthy and diseased subjects as well as in vitro and animal studies have suggested that different wavelengths [28,90,91,92], power and dose densities [93,94,95], and pulse frequencies [83,96] influence outcomes. With this state of knowledge, we can conclude that more work is needed to narrow down effective parameters in the quest for better applications and outcomes. In addition, the Inverse Square Law suggests that the distance between the light source (laser or LED) and the target surface should influence landed/irradiated power [97], and the position of the light source on the head, such as the hubs of the default mode network [98], could influence neurological outcomes.

With this background, further research on effective parameters could include the following:

1. Extend the investigation on tubulin polymerization [86] using a spread of different parameters.
2. Extend the investigation with Raman spectroscopy [87] covering a wide range of parameters.
3. Extend the EEG investigation using gamma at 40 Hz [83], alpha at 10 Hz, theta/delta at 4 Hz and other frequencies. In addition, we can seek real-time EEG readings for a better understanding of pulse frequency effects on brain waveforms and functions.
4. Measure the real-time response of the brain to various PBM parameters using fMRI. The precedence has been set with a real-time fMRI study by Nawashiro et al. published in 2017 on four cases. It demonstrated regional blood oxygen level dependency (BOLD) increases with laser at 810 nm wavelength, 204 mW/cm^2^ power density, and continuous wave for 90 s on and 60 s off for 3 times [99]. In 2020, Dmochowski et al. published a real-time fMRI study using a laser with 808 nm wavelength, 318 mW/cm^2^ power density, continuous wave, and 10 min duration on 20 subjects [100] The BOLD response in this study was more significant than that in Nawashiro et al. The difference in the level of response could be due to the treatment time. These studies can lead to new studies to determine whether applying different parameters such as wavelength, pulse frequencies, and light source positioning on the head will make a difference.
5. The efficacy of interventions for TBI is challenged by factors such as TBI’s heterogeneity and the variability in brain states and structures. Moreover, PBM presents a range of interventional parameters that can impact outcomes. The key to determining the most effective treatment may reside in a methodology involving iterative cycles of feedback and the careful selection of parameters from a wide array of choices. Incorporating artificial intelligence (AI) into this methodology could greatly expedite the process, enhancing the ability to personalize and optimize outcomes for individual patients.

## 7. Limitations of the Study

The limited number of human clinical studies available, along with a lack of common basis factors, hinders the conduct of a meaningful quantitative or meta-analytical synthesis. Based on the existing literature, clinical outcomes have been inconsistent. This inconsistency may stem from the wide variety of device parameters and study methodologies employed. Although PBM is known to alter physiological markers, which might lead to clinical outcomes, current data are insufficient to establish a general set of parameters that consistently predict outcomes with high confidence. The idea that personalizing treatment by adjusting pulse frequency, wavelengths, and other parameters can enhance effectiveness is primarily based on limited peer-reviewed research and preliminary data from ongoing studies. These ongoing studies are not yet published or peer-reviewed, and the discussions in this study include insights from the author’s forward-looking perspective.

## 8. Conclusions

This study proposes that photobiomodulation (PBM) can effectively modulate the pathophysiology of traumatic brain injury (TBI). PBM research can be aligned with the various pathophysiological aspects, highlighting its potential influence. The aspects include axonal injury, excitotoxicity, mitochondrial dysfunction, oxidative stress due to reactive oxygen species, neuroinflammation, axonal degeneration, growth inhibition, apoptotic cell death, and dysfunctional autophagy.

These insights collectively underscore PBM’s potential as a versatile treatment modality for TBI, applicable across different stages (acute or chronic) and severities, including chronic traumatic encephalopathy (CTE).

Available human clinical studies have corroborated PBM’s potential in TBI treatment. However, there is considerable variability in the parameters used in these studies, such as wavelength, power, light source positioning, pulse frequency, and dosage. Our analysis indicates that each parameter can distinctly influence treatment outcomes. While not yet conclusively established, factors like the distance and positioning of the light source can also affect therapeutic results.

The current research indicates that our understanding of PBM’s full potential in treating TBI is still in its early stages. Intensifying research efforts and incorporating Artificial Intelligence (AI) could significantly advance our knowledge of the optimal PBM parameters, thereby enhancing treatment outcomes in TBI therapy.

In summary, transcranial PBM represents a multifaceted therapeutic intervention for TBI with vast potential which may be fulfilled by optimizing the parameters. Future research should investigate optimizing these parameters, which is possible by incorporating AI.

## Figures and Tables

**Table 2:** Summary of human TBI studies treated with PBM.

Study (Publication Year)	PBM Parameters	Results and Clinical Outcomes
Naeser et al. (2011) [75]	LEDs: 870 nm, 633 nm, 22.2 mW/cm^2^, continuous wave	Two case reports: improved focus, cognition, memory, and inhibition accuracy.
Naeser et al. (2014) [76]	LEDs: 870 nm, 633 nm, 22.2 mW/cm^2^, continuous wave	11 subjects, open protocol: improved sleep, reduced PTSD symptoms, and improved functions.
Hesse et al. (2015) [77]	Laser diodes: 785 nm lasers, 10 mW/cm^2^, 36, 5 Hz pulsing	5 patients with disorders of consciousness: improved alertness and awareness and potential epileptic fits.
Hipskind et al. (2018) [78]	LEDs: 629 nm, 850 nm, 6.7 mW/cm^2^, various pulsing rates	12 military veterans with chronic cases, clinical assessment, and SPECT imaging: improved cognitive function, neuropsychological scores, and regional cerebral blood flow.
Figueiro Longo et al. (2020) [74]	LEDs: 810 nm, 36 mW/cm^2^, continuous wave	68 subjects with moderate TBI in a randomized, double blind study: safety with no confirmed adverse events, indication of significant axonal remyelination at the 3-month time-point using diffusion MRI.
Chao et al. (2020) [54]	LEDs: 810 nm LEDs, intranasal and transcranial modules up to 100 mW/cm^2^, 10 Hz and 40 Hz	A single case of chronic TBI imaged with MRI: neurogenesis in chronic TBI recovery; improved connectivity, cerebral perfusion, and neuropsychological test scores.
Rindner et al. (2022) [79]	Lasers: 1064 nm, 250 mW/cm^2^, continuous wave	11 cases diagnosed with TBI: safe with potential cognitive and emotional benefits.
Chan et al. (2022, preprint) [80]	LEDs: 830 nm, 29 mW/cm^2^, continuous wave	Data analysis of 38 patients with moderate TBI in a double-blind study, using fMRI: changes in resting-state connectivity were observed, but the symptom changes were not significantly different from placebo.
Naeser et al. (2023) [8]	LEDs: 810 nm, up to 100 mW/cm^2^, 40 Hz	4 ex-NFL players with suspected CTE: significant improvements in PTSD, depression, pain, sleep, and brain network connectivity.
Liebel et al. (2022 poster, 2023 peer review pending) [81]	LEDs: 810 nm, up to 100 mW/cm^2^, 40 Hz	49 former athletes with repetitive TBI history: reductions in depression, PTSD, and adjustment symptoms Sleep quality, simple reaction time, and hand grip strength improved.
